# OrthoFinder: solving fundamental biases in whole genome comparisons dramatically improves orthogroup inference accuracy

**DOI:** 10.1186/s13059-015-0721-2

**Published:** 2015-08-06

**Authors:** David M. Emms, Steven Kelly

**Affiliations:** Department of Plant Sciences, University of Oxford, South Parks Road, Oxford, OX1 3RB UK

## Abstract

Identifying homology relationships between sequences is fundamental to biological research. Here we provide a novel orthogroup inference algorithm called OrthoFinder that solves a previously undetected gene length bias in orthogroup inference, resulting in significant improvements in accuracy. Using real benchmark datasets we demonstrate that OrthoFinder is more accurate than other orthogroup inference methods by between 8 % and 33 %. Furthermore, we demonstrate the utility of OrthoFinder by providing a complete classification of transcription factor gene families in plants revealing 6.9 million previously unobserved relationships.

**Electronic supplementary material:**

The online version of this article (doi:10.1186/s13059-015-0721-2) contains supplementary material, which is available to authorized users.

## Background and rationale

Identifying homology relationships between sequences is fundamental to all aspects of biological research. In addition to the pivotal role these inferences play in furthering our understanding of the evolution and diversity of life, they also provide a coherent framework for the extrapolation of biological knowledge between organisms. In this context, orthology inference underpins genome and transcriptome annotation and provides the foundation on which synthetic and systems biology is built. Given the importance of this process to biological research there has been a rich heritage of methodology development in this area with the production of several effective orthology databases and algorithms.

The most widely used methods for orthology inference can be classified into two distinct groups. One group of methods approaches the problem by inferring pairwise relationships between genes in two species, and then extending orthology to multiple species by identifying sets of genes spanning these species in which each gene-pair is an orthologue. Popular methods that adopt this approach include MultiParanoid [1] and OMA [2]. A confounding factor to these approaches is that gene duplications cause orthology relationships that are not one-to-one [3] and so orthology is not a transitive relationship (for example, if gene A is an orthologue of gene B, and gene B is an orthologue of gene C, it is not necessarily true that gene A is an orthologue of gene C) [4]. This lack of transitivity means that to capture all pairwise orthology relationships individual genes must be allowed to be members of more than one set [2], or the gene sets must be restricted to subsets of species that share the same last common ancestor [1]. Methods that adopt these pairwise approaches have high levels of precision in recovering orthologues, however, they suffer from low rates of recall in discovering the complete orthogroup due to these complications arising from gene duplications.

The second group of methods do not adopt this pairwise strategy but rather attempt to identify complete orthogroups; an orthogroup is the set of genes that are descended from a single gene in the last common ancestor of all the species being considered [2, 5–9]. Here an orthogroup by definition contains both orthologues and paralogues, and in this context is frequently used as a unit of comparison for comparative genomics [10–12]. In this work we follow this latter approach as it is a logical extension of orthology to multiple species. The most widely used orthogroup inference method is OrthoMCL [13] (usage assessed by citations n = 870 Scopus citations at the time of writing this article). OrthoMCL uses BLAST [14] to compute sequence similarity scores between sequences in multiple species and then uses the MCL clustering algorithm [15] to identify highly-connected clusters (groups of highly similar sequences) within this dataset.

In addition to the approaches discussed above, several methods have also been developed that incorporate gene synteny/co-linearity information to assist in the inference of orthogroups [16, 17]. For groups of organisms such as the Kinetoplastids, where gene synteny/co-linearity is well conserved [18] it can provide valuable additional information. However, synteny is not conserved over large evolutionary distances and thus can provide little assistance to the identification of related genes between distantly related groups such as plants and metazoa. Moreover, synteny is unavailable for *de novo* assembled transcriptomes and for fragmented, low-coverage genome assemblies. Thus there is a need to have accurate methods of orthogroup inference that do not require gene synteny information.

Here we present OrthoFinder, a novel method that infers orthogroups of protein coding genes. It is fast, easy to use and scalable to thousands of genomes. In tests using real benchmark datasets OrthoFinder outperforms all other commonly used orthogroup inference methods by between 8 % and 33 %. We further demonstrate the utility of OrthoFinder through the inference and analysis of plant transcription factor orthogroups. Here we use phylogenetic methods to validate the orthogroups and show that using OrthoFinder to infer orthogroups identifies millions of previously unobserved relationships. Further information about the algorithm can be found at [19] and a standalone implementation of the algorithm is available under the GPLv3 licence at [20].

### Problem definition, method evaluation and comparison to other approaches

#### Gene length bias in BLAST scores affects the accuracy of orthogroup detection

**Fig. 1:**
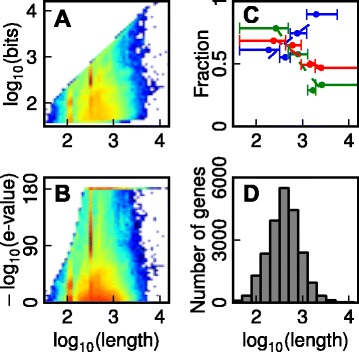
Analysis of gene length dependency of BLASTp scores. **a** BLAST log_10_(bit score) for all hits between *Homo sapiens* (Homo_sapiens.GRCh37.60.pep.all, 21,841 sequences) and *Mus musculus* (Mus_musculus.NCBIM37.60.pep.all, 23,111 sequences)*.* **b** –log_10_(e-value) for all hits between and *Homo sapiens* and *Mus musculus*. To avoid infinite values, BLAST scores of zero have been replaced with the lowest obtainable value 10^−180^. The heat map in both cases goes from blue (lowest density of hits) to red (highest). **c** The F-score (red), recall (blue) and precision (green) of orthogroup inference using OrthoMCL plotted as a function of sequence length. The sequences were sorted according to length and divided into four bins with the same number of sequences in each. The F-score, recall and precision were calculated for each bin and the scores plotted against the geometric mean of the length of the sequences in each bin. The error bars show the lower and upper limits of sequence lengths for the shortest and longest sequences in each bin and the dot shows the geometric mean of these lengths. **d** Histogram of all protein-coding gene lengths in *Homo sapiens* is provided for reference

The inference of orthogroups across multiple species requires a fast method to measure pairwise sequence similarity between all sequences in the species being considered. BLAST [14] is the most widely used method to identify and measure similarity between sequences and thus it underpins the majority of orthologue identification methods [9, 13, 21–23]. Analysis of the pairwise BLAST scores that are produced when the full set of protein sequences from one species is BLAST searched against those from another species revealed that there is a clear length dependency in the scores that are obtained (Fig. 1a and b). Short sequences cannot produce large bit scores or low e-values (Fig. 1a and b, respectively), whereas long sequences produce many hits with scores better than those for the best hits of short sequences (Fig. 1a and b). Thus, methods that construct orthogroups by evaluation of BLAST scores in the absence of gene length information should result in a large number of missing genes (low recall) from orthogroups that contain short genes and a large number of incorrectly clustered genes (low precision) in orthogroups that contain long genes.

To determine if this was the case we assessed the performance of OrthoMCL using the OrthoBench dataset [5]. OrthoBench is the only publicly available benchmark dataset of manually curated orthogroups. The dataset consists of 70 orthogroups of protein coding genes covering 12 species within the Metazoa where each orthogroup contains all the genes derived from a single gene in the last common ancestor of the 12 species considered. For further details concerning the construction, species range and complexity of each orthogroup see [5]. The recall and precision of OrthoMCL was assessed as a function of gene length in this dataset. This revealed that there were strong dependencies between the performance characteristics of OrthoMCL and the length of the gene that was being clustered (Fig. 1c, Additional file 1: Table S1). Specifically, short sequences suffer from low recall rate (that is, many short sequences fail to be assigned to an orthogroup) and long sequences suffer from low precision (that is, many long sequences are assigned to the incorrect orthogroup) as predicted from the analysis of BLAST scores above. To put these results in perspective the distribution of protein lengths in *Homo sapiens* is provided in Fig. 1d.

### A novel score transform eliminates gene length bias in orthogroup detection

Given that orthogroup inference shows a clear gene length dependency, we sought to develop a transform of the BLAST scores that would reduce the impact of gene length on clustering accuracy. To do this we developed a novel method that determines the gene length dependency of a given pairwise species comparison from an analysis of the bit scores from an all-versus-all BLAST search between the two species. Bit scores were used in place of e-values as the e-value calculation enforces a limit of 1×10^−180^ and thus all scores below this floor are given the same value (that is, 0) (Fig. 1b) and thus length bias in e-values is non-uniform and irreversible. As bit scores do not have a threshold value, and they have been previously shown to be capable of facilitating accurate inference of phylogenetic trees [24], they were selected as the raw data for the development of a novel score transform.

**Fig. 2:**
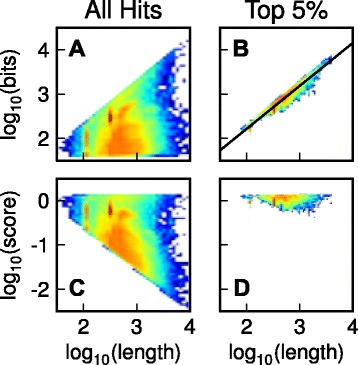
The gene length and phylogenetic distance normalisation procedure for a single species pair. **a** BLAST bit scores for all hits between *Homo sapiens* and *Mus musculus*. **b** BLAST bit scores for the top 5 % of BLAST hits with least-squares fit of the equation log_10_ *B*_*qh*_ = *a* log_10_ *L*_*qh*_ + *b*., where *B*_*qh*_ is the bit score for the hit between sequence q and sequence h and *L*_*qh*_ is the product of the gene lengths (measured in amino acids). **c** Gene length and phylogenetic distance normalised BLAST bit scores. Note that there are a large number of poor scoring hits for long sequences due to these hits exceeding the BLAST search e-value cutoff. **d** The same top 5 % of BLAST hits as shown in (b) after normalisation for reference

In brief, for each species-pair in turn, the all-vs-all BLAST hits (Fig. 2a) were divided into equal sized bins of increasing sequence length according to the product of the query and hit sequence lengths. The top 5 % of hits in each bin (ranked according to BLAST bit score) were used to represent ‘good’ hits for sequences of that length bin between the given species pair (Fig. 2b). A linear model in log-log space was used to fit a line to these scores using least squares fitting (Fig. 2b). All of the BLAST bit scores that were obtained from each species-pair all-vs-all BLAST search are then transformed using this model so that the best hits between sequences in this species pair have equivalent scores that are independent of sequence length (Fig. 2c and d). Following the transform the poor quality hits for longer sequences were no longer better than the best quality hits for short sequences (Fig. 2c). This normalisation procedure is applied to each pairwise species comparison independently as the behaviour of the BLAST scores is different for each pairwise species comparison (Additional file 2: Figure S1). Importantly, this pairwise length normalisation between species also normalises for phylogenetic distance between species (See ‘Gene length and phylogenetic distance normalisation’ & Additional file 2: Figure S1). Specifically, the normalisation ensures that the best scoring hits between distantly related species achieve the same scores (on average) to the best scoring hits between closely related species (Additional file 2: Figure S1). These length and phylogenetic distance normalised scores were then used as the measure of sequence similarity on which all subsequent analysis and clustering were performed.

**Fig. 3:**
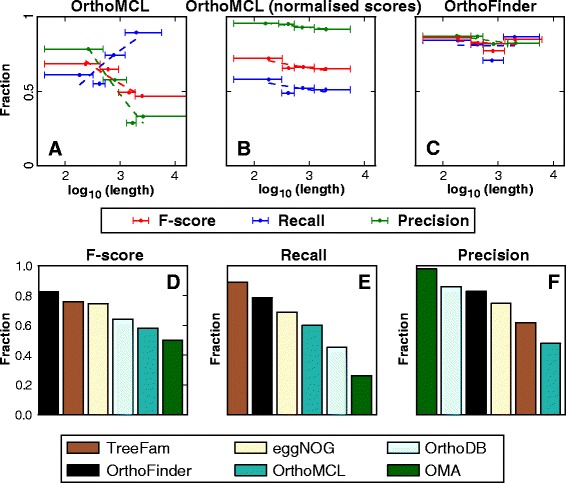
Comparison of OrthoFinder to other orthogroup inference methods. **a** The length dependency of OrthoMCL. **b** The length dependency of OrthoMCL using our normalised similarity scores. **c** The length dependency of the complete OrthoFinder algorithm. For A-C scores were calculated as in Fig. 1c. **d** Comparison of the results of OrthoFinder F-score with all other methods tested in OrthoBench. **e** As in (d) but for recall. **f** As in (d) but for precision. The error bars show the lower and upper limits of sequence lengths corresponding to the shortest and longest sequences in each bin and the dot shows the geometric mean of these lengths

Application of this novel score transform prior to clustering of the OrthoBench dataset resulted in a dramatic reduction in the length dependency of the clustering results (Fig. 3). Unlike OrthoMCL (Fig. 3a), neither precision, recall nor F-score displayed any dependency on gene length (Fig. 3b). Moreover, precision was substantially increased over the entire range of sequence lengths (Fig. 3b).

### An improved method for orthogroup delimitation improves overall accuracy

Given that we had reduced gene length bias and that precision was high but recall was low, we assessed whether a method that could identify a higher proportion of cognate gene-pairs prior to clustering could produce an overall increase in clustering accuracy. Many orthology assignment methods make use of reciprocal best BLAST hit (RBH) as it is widely regarded as a high precision method for the identification of orthologues gene-pairs [25–27]. Therefore we also sought to use reciprocal best BLAST hits using our new length-normalised score to assist in construction of the orthogroup graph. Henceforth, we refer to a reciprocal best hit that is obtained using the length-normalised score as an RBNH (reciprocal best normalised hit).

In brief, for each gene that had successfully identified one or more RBNHs, the scores for these RBNHs were used to delimit an inclusion threshold (see methods). As all scores are normalised for gene length and phylogenetic distance, hits to other genes (in any species) that had scores above this inclusion threshold were included as putative cognate gene-pairs and added to the orthogroup graph that was subjected to MCL clustering (for further details see methods). This novel data selection criterion resulted in a dramatic improvement in overall clustering accuracy while maintaining gene length independence (Fig. 3c). The overall results for OrthoFinder, were 0.85 precision, 0.81 recall and 0.83 F-score.

### OrthoFinder outperforms all other methods from the OrthoBench analysis

Given that OrthoFinder exhibited high accuracy on the benchmark dataset we sought to determine the relative performance to other commonly used methods for orthogroup inference. OrthoFinder outperformed all other methods that have been applied to OrthoBench [5] as measured by F-score (Fig. 3d), performing 8 % better than TreeFam (the next best method) 25 % better than OrthoMCL (the most widely used method), and 33 % better than OMA (the lowest scoring method in this test). Importantly, the precision and recall of OrthoFinder were balanced, demonstrating that the method is not biased towards over- or under-clustering of sequences. It should be noted that OMA exhibits a low recall in this test as its goal is to identify orthologues instead of complete orthogroups and thus paralogues will be absent from the orthologue groups identified by this method. OMA is included here for completeness as it was included in the original OrthoBench analysis [5].

In addition to accuracy, a number of other criteria were used to compare the performance of the different inference methods in the OrthoBench paper. These criteria included the percentage of orthogroups predicted without any errors, the number of erroneously assigned genes (that is, false positives, and thus also captured by the precision) and missing genes (that is, false negatives, and thus also captured by recall) in the assignment of genes to orthogroups and the proportion of orthogroups affected by these false positive and false negatives. The results for OrthoFinder according to these criteria are reported in Additional file 3: Figure S2 and are consistent with the increased accuracy of OrthoFinder compared to other methods. Additionally, the 70 orthogroups that make up the OrthoBench dataset comprise 40 that represent particular biological or technical challenges and 30 randomly chosen orthogroups. Additional file 4: Figure S3 shows the F-scores for these two categories separately to illustrate the difference in performance of the method for ‘randomly selected’ and ‘difficult’ orthogroups. OrthoFinder outperformed all other methods in both categories and achieved an F-score of 81 % and 90 % on the difficult and randomly selected orthogroups, respectively.

### OrthoFinder is suitable for the analysis of incomplete datasets

As many research groups are producing partial genome assemblies and transcriptome resources it is to be expected that sequence datasets will be missing genes due to incomplete assembly, low expression or errors in gene prediction. To demonstrate the suitability of OrthoFinder for analysing these incomplete datasets we assessed the performance of OrthoFinder with between 5 % and 60 % of genes deleted at random from the OrthoBench input sequences. This revealed that the accuracy of OrthoFinder is robust to missing data and that it achieved an F-score of over of 0.75 even when 60 % of the genes were missing from the input dataset (Additional file 5: Figure S4). Thus OrthoFinder is suitable for orthogroup inference from partial and incomplete datasets.

### OrthoFinder is fast and scalable

**Fig. 4:**
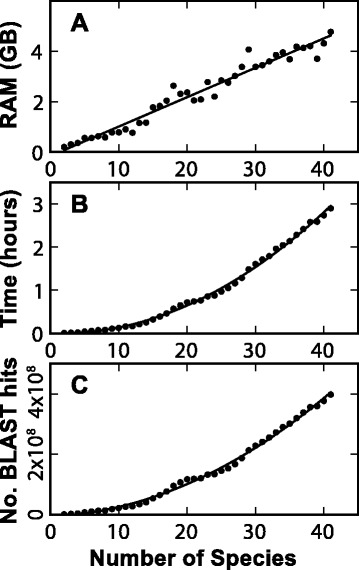
Memory and time requirements of OrthoFinder. Sub-samples of between two and 41 plant genomes from Phytozome version 9.0 given pre-calculated BLAST results. The average number of genes per species was 30,731. **a** Maximum RAM requirements. **b** Time requirements. **c** The number of BLAST hits that must be processed for a given number of species (provided to place the time and RAM requirements into context)

The number of species for which genome or transcriptome sequence resources are available is increasing rapidly and there is a corresponding need to be able to infer orthogroups using these datasets as they emerge. To keep pace with these increasing demands the algorithm utilises sparse matrices as the central data structure and performs many steps using matrix operations. For example, starting from pre-computed raw BLAST scores the identification of orthogroups for the OrthoBench dataset (12 species, 235,033 sequences) takes 14 min 20 s using OrthoFinder on a single core of an Intel Core i7-4770 3.4GHz CPU. For comparison, OrthoMCL takes 20 h 12 min to perform the same operation using the same CPU and MySQL for its relational database management system. As the number of genomes that must be analysed increases, the scalability of the methods used becomes increasingly important. To demonstrate the scalability performance of OrthoFinder, the full set of sequenced plant genomes from Phytozome version 9.0 (n = 41 [28]) were clustered and the results are shown in Fig. 4. Plant genomes were selected for this test as they are large with an average of 30,731 protein coding genes per species in Phytozome version 9.0 and thus they represent a stringent assessment of the scalability of OrthoFinder. The memory (RAM) requirements increase linearly with the number of species clustered (Fig. 4a). This is despite the fact that the number of BLAST hits increases quadratically with the number of species (Fig. 4c). This linear scaling is achieved by processing the BLAST hits for each species sequentially and independently within OrthoFinder. Though the memory requirements increase linearly, the time requirements starting from pre-computed raw BLAST scores increases quadratically with the number of species (Fig. 4b). This is to be expected as the number of BLAST hits that must be processed also increases quadratically. For example, identifying the orthogroups for all 41 plant species from Phytozome requires approximately 4 GB of RAM and took approximately 3 h on a single CPU core. Fitting the data to a line and extrapolating we estimate that approximately 450 plant sized genomes can be clustered on a linux computer with 64GB of RAM (Fig. 4a). Thus OrthoFinder is capable of large analyses on conventional computing resources. It should be noted here that the BLAST searches incur the largest computational cost in any orthogroup inference analysis and that this cost is the same for all inference methods that use BLAST. In summary OrthoFinder is fast and scalable to hundreds of species on conventional computing resources.

### Inference of high accuracy plant transcription factor orthogroups

Given that OrthoFinder has increased accuracy over other methods and that gene length bias has been eliminated from orthogroup inference, we sought to provide an additional demonstration of the utility of OrthoFinder for the inference of orthogroups. To do this we selected plant transcription factors as they are short genes and will thus suffer from low rates of recall in assignment to orthogroups in the absence of gene length bias correction. Moreover transcription factor genes are preferentially retained following whole genome duplication events [29, 30] and thus transcription factor orthogroups are larger than average and contain multiple independent duplication events in multiple independent lineages that can cause some inference methods to fail. Finally, previous efforts to define transcription factor orthogroups have utilised OrthoMCL [31]. Thus current transcription factor orthogroups will have low recall resulting in fragmented orthogroups spanning few species.

**Fig. 5:**
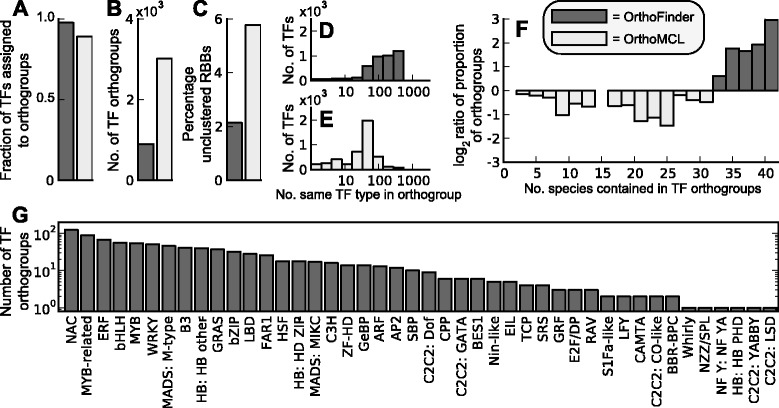
Inference of orthogroups of plant transcription factors. In all cases dark grey bars indicate the results for OrthoFinder and light grey bars indicate the results for OrthoMCL. **a** Comparison of the fraction of transcription factors that are assigned to orthogroups by OrthoFinder and by OrthoMCL. **b** Comparison of the number of transcription factor orthogroups identified using each method. **c** The percentage of RBNH/RBH (for OrthoFinder/OrthoMCL) hits that are not contained in orthogroups identified using each method. **d** The number of transcription factors of the same type that each transcription factor is connected to in the orthogroups produced by OrthoFinder. **e** as in (d) but for OrthoMCL. **f** Comparison of species coverage for transcription factor orthogroups identified by each method. **g** The number of orthogroups for each transcription factor type identified by OrthoFinder

Using established rules for the identification and classification of transcription factors [31] we identified and typed all of the transcription factors present in the 41 genomes present in Phytozome v9. The complete predicted proteomes from these 41 genomes were then subject to clustering using OrthoFinder and OrthoMCL and the distribution of transcription factors in the resultant orthogroups were analysed. OrthoMCL was used here as it is the method by which all transcription factor families are currently classified [31]. Consistent with the increased recall rate for OrthoFinder, analysis of the resulting orthogroups revealed that 8.5 % more transcription factors were placed in orthogroups using OrthoFinder than OrthoMCL (Fig. 5a, 97.6 % and 89.1 %, respectively, n = 52,744). Also consistent with the increased recall rate is that these orthogroups were less fragmented than those that were produced by OrthoMCL (Fig. 5b, 897 and 3,024 orthogroups, respectively). Importantly, the orthogroups inferred using OrthoFinder were missing fewer RBHs (Fig. 5c, 2.15 % and 5.77 %, respectively) and clustered more of the same type of transcription factor together (Fig. 5d and e). A major difference between those orthogroups inferred using OrthoFinder and OrthoMCL is that those produced by OrthoFinder encompass a larger number of species than those recovered by OrthoMCL (Fig. 5f), thus orthogroups produced by OrthoFinder encompass greater phylogenetic distances.

As OrthoFinder clustered the transcription factors together into far fewer orthogroups than OrthoMCL (897 versus 3024) we sought to demonstrate that it was correct in doing so. To do this we used gene-tree/species-tree reconciliation to determine if the orthogroups were true orthogroups if they incorrectly clustered sequences that are separated by a gene duplication event that occurred before the last common ancestor of the species in the analysis. Overall, 858 of the 897 OrthoFinder orthogroups (96 %) consisted entirely of genes that were correctly clustered together and only 39 contained some genes that were separated by a duplication prior to the last common ancestor (Additional file 6: Table S2 and Additional file 7: Table S3). Of the 897 OrthoFinder orthogroups, 210 were identical to ones from OrthoMCL and 471 OrthoFinder orthogroups were strict supersets of 2,271 OrthoMCL orthogroups (Additional file 6: Table S2 and Additional file 7: Table S3). Of these, 90 % (425) were true orthogroups that each encompassed on average four OrthoMCL orthogroups (1,709 in total).

**Fig. 6:**
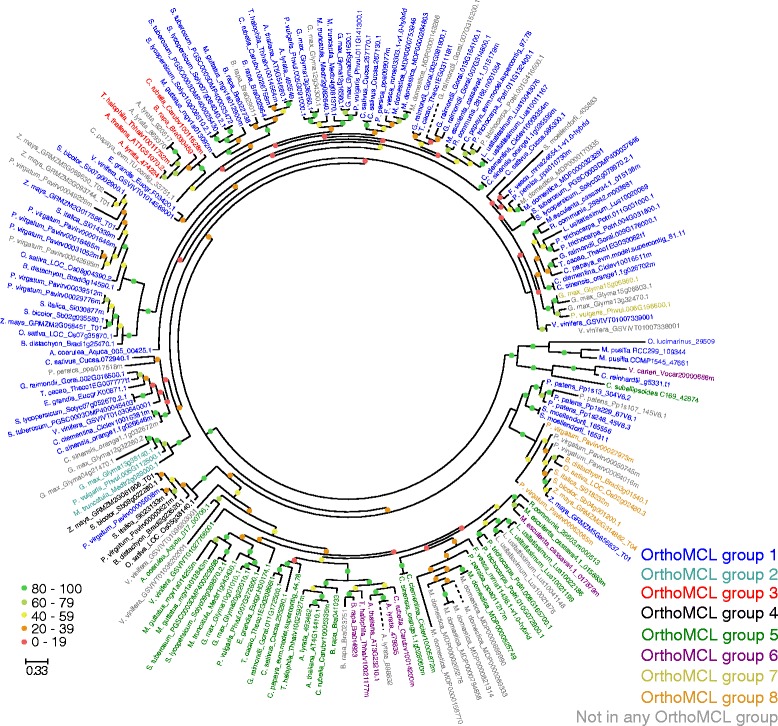
A bootstrapped maximum likelihood phylogenetic tree of the OrthoFinder orthogroup containing the type IVc bHLH transcription factors (bHLH 8). The OrthoMCL orthogroups that are subsets of the OrthoFinder orthogroup are indicated by different coloured fonts. Thirty-six of the OrthoFinder clustered genes (coloured grey) failed to be clustered in any OrthoMCL orthogroup. The tree was inferred using RAxML using the PROTGAMMAAUTO model (the JTT was model was selected as having the highest likelihood) with 100 bootstrap replicates. Scale bar indicates the number of substitutions per site. Percentage bootstrap support values are indicated by coloured circles shown at internal nodes

An illustrated example showing an OrthoFinder orthogroup and its constituent OrthoMCL orthogroups is provided in Fig. 6. Here the OrthoFinder orthogroup (labelled bHLH 8 in Additional file 6: Table S2) contains all known type IVc bHLH transcription factors [32]. Type IVc bHLH transcription factors have previously been shown to be conserved from green algae to land plants and thus span the complete taxonomic range contained in this analysis [32]. The OrthoFinder orthogroup correctly united eight paraphyletic OrthoMCL orthogroups and included 36 transcription factors (highlighted in grey) that were not clustered into any orthogroups by OrthoMCL (Fig. 6). The phylogenetic tree shows that there are no genes present in this OrthoFinder orthogroup that were the product of a gene duplication event prior to the divergence of the last common ancestor of all species in the analysis. This is only one example and the complete set of phylogenetic trees for each OrthoFinder transcription factor orthogroup are provided in Additional file 6: Table S2 along with the OrthoMCL subsets that comprise these groups where appropriate. Also contained in this table are the results of the gene-tree/species-tree reconciliation for each tree inferred from an OrthoFinder orthogroup.

Taken together, using OrthoFinder to cluster transcription factor genes resulted in the identification 687 (897 less the 210 that were the same) novel orthogroups of transcription factors across 41 different species comprising 7.7 million pairwise relationships (of which 6.9 million are not detected by OrthoMCL). Thus using OrthoFinder to cluster transcription factors has provided significant new insight into the relationship of transcription factor genes across plants. The number of orthogroups for each transcription factor type is provided in Fig. 5g and the full classification including all constituent accession numbers is provided in Additional file 6: Table S2.

### Algorithm implementation and evaluation criteria

OrthoFinder is an algorithm that infers orthogroups across multiple species. The method does not classify the pairwise relationships that exist between genes within these orthogroups. The method does not require synteny information and is thus equally suitable for clustering protein sequences predicted from genome or transcriptome datasets. OrthoFinder is run with a single command and requires as input a directory containing one protein sequence FASTA file per species to be clustered. OrthoFinder does not require preprocessing of FASTA files (such as filtering of sequences) and does not require knowledge or use of any relational database management system such as MySQL. It outputs orthogroups in two file formats: the *Quest for Orthologs* community standard OrthoXML [33] and in plain text format with one orthogroup per line.

There are two common problem definitions used by the majority of homology inference algorithms. One is to predict pairs of orthologues (pairs of genes from two different species descendent from a single gene in the last common ancestor of the two species) and pairs of recent, within-species paralogues (genes-pairs arising from a duplication event since the last speciation event for that species). The other approach, and the one used here for OrthoFinder, is to predict orthogroups. An orthogroup is the set of genes derived from a single gene in the last common ancestor of all the species under consideration. This is the approach used by OrthoMCL [13] and eggNOG [34]. OrthoFinder follows this second approach to produce orthogroups of protein coding genes as this is a logical extension of orthology to multiple species as it groups all genes descended from a single gene in the last common ancestor of all species being considered.

### Methodological overview of the OrthoFinder algorithm

**Fig. 7:**
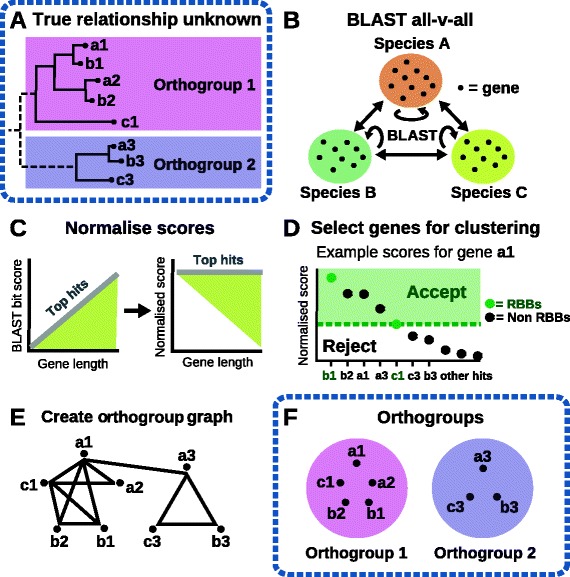
Overview of the steps in the OrthoFinder algorithm for two example orthogroups of genes from three species. **a** The unknown orthogroups that the algorithm must recover, shown as a gene tree. **b** BLAST search of all genes against all genes. **c** Gene length and phylogenetic distance normalisation of BLAST bit scores to give the scores to be used for orthogroup inference. **d** Selection of putative cognate gene-pairs from normalised BLAST scores. **e** Construction of orthogroup graph, genes are nodes in the graph and pairs of genes are connected by an edge with edge weights given by the normalised bit score. **f** Clustering of genes into discrete orthogroups using MCL

An overview of the algorithm in shown in Fig. 7, it proceeds in five phases corresponding to sections b-f in the figure:

1. **BLAST all-versus-all search (Fig.****7b****).** Protein BLAST (blastp) with an e-value threshold of 10^−3^ is used so as to avoid discarding putative good hits for very short sequences. A relaxed threshold is used at this stage of the method as subsequent steps filter out false positive hits using stringent, orthogroup-specific criteria for inclusion (described below).
2. **Gene length and phylogenetic distance normalisation of the BLAST bit scores (Fig.****7c****).** This step models the all-vs-all BLAST hits for each pairwise comparison between species to identify and remove the gene similarity dependency on gene length and phylogenetic distance. This is done so that the best hits between all species achieve the same scores regardless of sequence length or phylogenetic distance.
3. **Delimitation of orthogroup sequence similarity thresholds using RBNHs (Fig.****7d****).** This step uses information from RBNHs (Reciprocal Best length-Normalised hit) to define the lower limit of sequence similarity for putative cognate genes of each query gene. To be included in the orthogroup graph a gene-pair must be an RBNH or produce a hit that is better scoring than the lowest scoring RBNH (irrespective of species) for either gene.
4. **Constructing an orthogroup graph for input into MCL (Fig.****7e****).** Putative cognate gene-pairs are identified as above and are connected in the orthogroup graph with weights given by the normalised BLAST bit scores.
5. **Clustering of genes into orthogroups using MCL (Fig.****7f****)**.

The steps 2 to 4 are the novel parts of our algorithm and are described in detail below.

### Gene length and phylogenetic distance normalisation

The aim of this normalisation procedure is to remove gene length bias from BLAST bit scores and to normalise for phylogenetic distance between species. MCL converts sets of similarity scores into clusters by breaking apart clusters of genes that have low similarity scores (and therefore are unlikely to be orthogroups) and preserving clusters of sequences that have high similarity scores. If the similarity scores between long sequences are inherently larger than the similarity scores between short sequences then the clustering will preferentially break apart clusters of short sequences while preserving clusters of long sequences. This effect can be clearly seen in the results of a typical OrthoMCL cluster. Here, long sequences are placed in overly large clusters leading to low precision, and short sequences remain un-clustered leading to low recall (Fig. 3a). The species-wise normalisation implemented by OrthoFinder similarly ensures that orthologues from more distant species (that have inherently lower similarity scores due to phylogenetic distance) are not preferentially discarded and is similar to a step that is performed in OrthoMCL wherein all scores are divided by the average score between a given species pair [13].

Previous methods have exploited BLAST e-values (rather than bit scores) as a measure of similarity between sequences. However, as can be seen in Fig. 1b the use of e-values for assessment of similarity between sequences is flawed. Here, the minimum e-value that can be obtained for a given query sequence decreases with increasing sequence length until, at a certain length, the lower bound for e-values is reached and BLAST returns an e-value of 0. This creates two problems: (1) long sequences will frequently have low quality hits with better e-values than the best possible hits of short sequences; and (2) the floor value for the e-value calculation means that length bias is non-uniform and thus irreversible. Specifically, beyond the floor-value e-values cannot be used to distinguish between the qualities of hits as they are all assigned the same e-value. As can be seen in the heat map shown in Fig. 1b, many hits obtain this floor-value for a given pairwise species comparison and thus their similarities are indistinguishable. This length-bias must be removed to prevent biasing downstream clustering applications.

In this method we construct a similarity measure between sequences based on the bit-score normalised to take into account the query and hit sequences lengths and the phylogenetic distance between species. Unlike e-values, the bit-scores do not suffer from the presence of a threshold limit and thus different amounts of sequence similarity can be distinguished regardless of the lengths of the sequences involved. Let *L*_*q*_ be the length of the query sequence and *L*_*h*_ be the length of the hit sequence. In an analogous manner to the e-value calculation made by BLAST and other sequence comparison methods, we use the variable *L*_*qh*_ = *L*_*q*_*L*_*h*_ to quantify the lengths of a pair of sequences that are being compared.

The length normalisation procedure is shown in Fig. 2. For each species pair, we:

1. Sort all BLAST hits according to *L*_*qh*_ = *L*_*q*_*L*_*h*_.
2. Put the hits into equal sized bins of 1,000 hits (put the ‘shortest’ 1,000 hits according to *L*_*qh*_ into one bin, the next 1,000 hits into the next bin and so on for all the hits). If there are fewer than 5,000 hits then we put the hits into bins of 200. Using fixed sized bins means that it is not necessary for the algorithm to specify the location of the bins in advance.
3. Sort the hits in each bin according to BLAST bit score and select the top 5 % of hits from each bin. Find the parameters a and b that best describe the fit between sequence similarity scores and gene length for the selected hits using the equation log_10_*B*_*qh*_ = *a* log_10_*L*_*qh*_ + *b* where *B*_*qh*_ is the BLAST bit score between sequences q and h.
4. Normalise all obtained BLAST bit scores (not just the top 5 %) between the given species pair according to, *B*_*qh*_^'^ = *B*_*qh*_/10^*b*^*L*_*qh*_^*a*^, so that *B*^'^_*qh*_, (the normalised score) is the BLAST bit score for a hit divided by the BLAST bit score that would be expected for the best hits between sequences of that length for the species pair under consideration.

The top 5 % of hits are used rather than RBHs as selection of RBHs will be affected by the gene length-bias that we wish to correct. Moreover, gene duplication events can frequently cause RBHs to fail (Additional file 8: Figure S5) and thus reduce the number of data points that are available for fitting. The normalisation procedure ensures that the best hits between a given species pair achieve (on average) the same scores irrespective of their gene length.

OrthoFinder also normalises for phylogenetic distance, this is done so that the similarity scores between orthologues will be independent of phylogenetic distance (that is, the true orthologues in distantly related species will obtain similar scores to the true orthologues in closely related species). If this step is not done then true orthologues in distantly related species will always obtain lower scores than true orthologues in closely related species. Thus during graph clustering (which is unaware of phylogenetic relationship between species) distantly related true orthologues (and cognates) will become disconnected from each other more easily than closely related true orthologues (and cognates) in the orthogroup graph. Previous efforts to prevent this phylogenetic bias include dividing the observed similarity score for any given gene-pair by the mean similarity score observed for all reciprocal best hits between genes in that species pair [13]. However, in the absence of gene length information this means that short genes will always be penalised more than long genes.

Though there is precedent for the use of *L*_*qh*_ = *L*_*q*_*L*_*h*_ to quantify the lengths of a pair of sequences that are being compared [14], different functions for gene length normalisation were also assessed. All other functions, including for example the use of the variable ${\tilde{L}}_{qh}={L}_q+{L}_h$ in place of *L*_*qh*_, gave a lower overall clustering accuracy.

### Identification of putative cognate gene-pairs for inclusion in the orthogroup graph

Once scores are normalised OrthoFinder exploits RBNHs to identify putative cognate gene-pairs. RBHs are a high precision method to identify putative orthologues [25–27] and OrthoFinder uses the reciprocal requirement exploiting its length and phylogenetic distance normalised BLAST scores. For each gene the scores for its RBNHs are used to delimit the extent of sequence similarity of that gene’s orthogroup. Specifically, for each query sequence, q, any hit, h, with a normalised score, *B*^'^_*qh*_, greater than or equal to the score for the lowest scoring RBNH of q is selected as a putative cognate gene-pair of q and therefore is connected to q in the orthogroup graph that is subsequently subjected to MCL clustering.

The rationale for this approach is that the level of normalised similarity of a query gene and its RBNHs can be used to estimate the extent of similarity of other genes within the same orthogroup. All genes more similar to a query gene than any of the query gene’s RBNHs (irrespective of species) are likely members of the same orthogroup. Therefore, the normalised similarity score for the most dissimilar RBNH of a gene is used as a cutoff for inclusion of additional cognate gene-pairs from all species. That is q is connected to h in the orthogroup graph if *B*^'^_*qh*_ > *B*^'^_*qR*_ where R is an RBNH of q. This provides a simple and robust method for recovering cognate gene-pairs that may otherwise be difficult to detect due to duplication events that can cause the RBNH method to fail. Further details, explanation and worked examples are provided in Additional file 8: Figure S5.

In summary, the novel method presented here generates, for each query gene, an independent prediction of all the genes in its orthogroup. This orthogroup graph is then clustered using MCL with its default inflation parameter of 1.5. The effect of varying the MCL inflation parameter on the OrthoFinder result is shown in Additional file 9: Figure S6. The F-score of OrthoFinder is relatively stable to variation in MCL inflation parameter, however it is possible to trade precision against recall by varying this parameter (Additional file 9: Figure S6). For comparison the analogous analysis is also presented for OrhtoMCL (Additional file 10: Figure S7).

### Implementation

OrthoFinder is written in python. It requires python together with the numpy and scipy libraries [35] to be installed. OrthoFinder requires the standalone BLAST+ and MCL algorithms that are freely available. These standalone applications must be installed separately to OrthoFinder and are not included in the OrthoFinder package. The implementation makes use of sparse matrices to store hits between sequences. This provides a memory efficient method of storing the data and allows key parts of the algorithm to be expressed using scipy’s highly optimised C++ implementations of sparse matrix operations. OrthoFinder can either run the BLAST searches for you or can be run on pre-computed BLAST searches. If you chose to run BLAST searches independently then instructions are provided in the documentation for how to process your sequence names in the pre-computed BLAST output. Similarly OrthoFinder will also automatically run MCL for you. However if you wish to run MCL separately using different parameter settings then the MCL input files are stored for this purpose in a working directory.

### Evaluation

OrthoBench [5] is the only manually curated dataset of orthogroups for the assessment of orthogroup prediction algorithms. It was used in this work for assessing OrthoFinder as it has been independently evaluated, it underpins the testing of multiple different methods and it is a well-defined and stringent test of the problem that OrthoFinder was designed to solve. Criteria such as functional similarity within orthogroups, expressed for example using enzyme classification numbers [36], were not used in this work since not all proteins with the same function are members of the same orthogroup and members of the same orthogroup do not necessarily all have the same function. As we are using real benchmark datasets for which only a subset of sequences have been assigned to ‘true’ gene families the extent of true negative orthologue assignments is unknown (as is the case for all methods tested on this dataset). Thus we cannot use the Matthews correlation coefficient to assess the performance of the orthogroup inference methods. In the absence of this information the simplest and most transparent evaluation of the accuracy of any prediction method is to measure its precision and recall.

$$ precision=\frac{TP}{TP+FP} $$

$$ recall=\frac{TP}{TP+FN} $$

Where TP is the number of true positive orthogroup assignments (that is, correct assignments), FP is the number of false positive orthogroup assignments (that is, incorrect assignments) and FN is the number of false negative orthologue assignments (that is, missing assignments). The F-score is the harmonic mean of these two measures, where the harmonic mean weights towards the worst performing measure. We also provide other evaluation measures from the original OrthoBench analysis in Additional file 3: Figure S2.

### Inference of transcription factor orthogroups

To infer transcription factor orthogroups we first identified the set of transcription factors present in all genomes present in Phytozome V9. This identification was performed using the same rules for the presence and absence of PFAM domains as has been previously described [31]. The full set of protein coding genes from these genomes (including all the transcription factors) was then clustered using OrthoFinder and OrthoMCL and the distribution of the transcription factors within these orthogroups was analysed. OrthoMCL was selected for comparison here as it is the method by which all orthogroups of transcription factors are currently defined [31]. An orthogroup of transcription factors was defined as an orthogroup whose constituent genes comprised ≥50 % transcription factors of the same domain classification.

To determine if OrthoFinder was correct in combining multiple separate OrthoMCL orthogroups each orthogroup was subject to gene-tree—species-tree reconciliation. Using, gene-tree species-tree reconciliation it is possible to determine if OrthoFinder had incorrectly placed together genes that had diverged prior to the last common ancestor of the species being analysed. To do this, gene trees were inferred for each orthogroup by aligning the sequences using mafft-linsi [37] and inferring a maximum likelihood tree from this alignment using FastTree [38]. DLCpar [39] was used to reconcile these gene trees with the known species tree [28]. Using this method, each gene tree was assessed to determine if it contained bipartitions that occurred prior to the divergence of the last common ancestor of all the species being analysed. If such a bipartition was identified then the orthogroup was considered not to be a true orthogroup as it contained one or more genes that evolved by duplication prior to the last common ancestor of all species under consideration.

## Discussion

In this work we have presented OrthoFinder, a novel method for inference of orthogroups. Our method is focused on a clear definition of an orthogroup, that is, that an orthogroup contains all genes descended from a single gene in the last common ancestor of the species whose genes are being analysed. This definition avoids conflating shared ancestry with other criteria that are not equivalent, such as functional conservation. Our method is designed to address the problem of orthogroup inference rather than categorise the disparate relationships that occur between individual genes within an orthogroup. These relationships are best resolved by first inferring orthogroups using OrthoFinder and then using multiple sequence alignment and phylogenetic methods on these orthogroups.

The two key novel features of our method are: (1) a method to automatically remove gene length bias and phylogenetic distance from sequence similarity scores; and (2) a novel method to define the sequence similarity limits of an orthogroup. In the tests performed on the only publicly available orthogroup benchmark dataset (OrthoBench) OrthoFinder outperformed all of the commonly used orthogroup assignment methods by between 8 % and 33 %. Moreover we have shown OrthoFinder to be scalable and robust to missing genes typical of incomplete genomes and *de novo* transcriptome assemblies. The software is freely available and can take pre-computed BLAST scores as input making it easy to test on any newly developed benchmarks for which pre-computed BLAST scores are available.

We further demonstrate the utility of OrthoFinder by providing a novel classification of all transcription factors in the available, fully-sequenced plant genomes present in Phytozome V9. This analysis clusters 97.6 % of the 52,744 putative transcription factors into orthogroups. This novel analysis identifies millions of relationships that have not previously been reported providing new insight into the relationship and evolution of transcription factor gene families in plants.

Inferring orthologues underpins much of modern biological research and is among the first steps in the annotation and analysis of genome and transcriptome sequencing projects. As sequencing technologies are now within the budgets of most research groups these data resources are increasing in number rapidly. Thus there is a requirement for an orthogroup inference method that is accurate, robust, scalable, and that can be run easily by independent research groups on conventional computing resources. Many orthogroup inference methods are not available for general use but are provided as static databases (for example, EggNog and TreeFam). Thus the most widely used methods are those that enable researchers to analyse their own data resources. With this in mind OrthoFinder has been developed with the aim of being easy to use. The method is executed as a single command, has minimal dependencies and requires as input just the individual protein sequence FASTA files for each species that is being clustered. The algorithm carries out all calculations (including BLAST searches and MCL clustering) and outputs the orthogroups in both a plain tab delimited text file and in the OrthoXML community format. The algorithm itself is small, fast and memory efficient, making it suitable for use on linux desktop computers. Further information about the algorithm can be found at [19] and a standalone implementation of the algorithm is available under the GPLv3 licence at [20].

## Additional files

Additional file 1: Table S1. Table of all false positive and false negative genes produced by clustering OrthoBench using OrthoMCL. (XLSX 57 kb) Additional file 2: Figure S1. An overview of how the OrthoFinder score transform also normalises for phylogenetic distance between BLAST scores. For illustration the All-Vs-All BLASTp scores are shown for the longest protein isoform from each protein coding gene in *Homo sapiens* vs all other species in the test. Note the difference in properties of fitted line, here the slope of the lines for the more closely related species are greater than for the more distant species. Following transform all fitted lines are transformed to the same value with a slope of 0. Thus the best scoring hits between distantly related species pairs and closely related species pairs achieve the same score hence normalising for interspecies phylogenetic distance. To provide further illustration all fitted lines between *Homo sapiens* and all other species before and after normalisation are shown on the right. (PDF 1440 kb) Additional file 3: Figure S2. Results on the OrthoBench dataset using additional assessment criteria presented in the original OrthoBench paper (the calculation for one of the plots could not be reproduced using the information provided in the OrthoBench paper and so has not been included). (PDF 29 kb) Additional file 4: Figure S3. F-scores on the OrthoBench dataset for the 30 randomly chosen gene families and the 40 biologically or technically challenging gene families that make up the dataset. (PDF 357 kb) Additional file 5: Figure S4. Accuracy of OrthoFinder as a function of fraction of missing sequences. With poor gene coverage many RBNBs will be missing and so cannot inform the identification of orthogroups. To simulate this, genes were removed at random from the OrthoBench dataset input into the OrthoFinder and the precision, recall and F-score on the remaining genes were measured. (PDF 356 kb) Additional file 6: Table S2. Transcription factor orthogroups inferred using OrthoFinder. Orthogroups are named according to the PFAM domain classification and from largest to smallest where #1 is the largest orthogroup of that transcription factor type. Accession numbers provided are correct for Phytozome version 9. Maximum likelihood phylogenetic trees are provided in column AS. The relationship of the OrthoFinder orthogroup to OrthoMCL orthogroups is listed in column B. ‘Identical’ means that the OrthoFinder orthogroup is identical to an OrthoMCL orthogroup. ‘Superset’ means that the OrthoFinder orthogroup is a superset of more than one OrthoMCL orthogroups. ‘Subset’ means that the OrthoFinder orthogroup is a subset of a larger OrthoMCL orthogroup. ‘Neither subset nor superset’ means that the OrthoFinder orthogroup contains sequences that were not clustered into any orthogroups by OrthoMCL. The results of the gene tree species tree reconciliation are provided in column C, this column specifics whether the phylogenetic tree inferred from the OrthoFinder orthogroup contained genes that were separated by a duplication prior to the last common ancestor. (XLSX 2110 kb) Additional file 7: Table S3. Results of the gene tree species tree reconciliation analysis. This table contains a summary of the full dataset presented in Additional file 1: Table S1. LCA is the last common ancestor of all the species being analysed. OG is orthogroup. (XLSX 10 kb) Additional file 8: Figure S5. A worked example showing the criteria for identification of putative cognate gene-pairs used by OrthoFinder. (PDF 72 kb) Additional file 9: Figure S6. The effect of the MCL inflation parameter on the F-score, precision and recall of OrthoFinder on the OrthoBench dataset. In OrthoFinder we use the default parameter of 1.5 which gives the results reported in this paper (84.5 %, 80.8 % and 82.6 % for precision, recall and F-score, respectively). Increasing the inflation parameter can be used to achieve higher precision at the cost of lower recall. Conversely, a smaller value of inflation can be used to achieve higher recall at the cost of lower precision. In this dataset the best result obtained by OrthoFinder in terms of F-score was 83.9 % using a value of 1.7 for the inflation parameter. The scores for precision and recall were 88.4 % and 79.8 %, respectively. (PDF 357 kb) Additional file 10: Figure S7. The effect of the MCL inflation parameter on the F-score, precision and recall of OrthoMCL on the OrthoBench dataset. The standalone OrthoMCL v2.0.9 was run on the OrthoBench dataset to produce the MCL input graph and MCL was rerun on this graph with a range of inflation parameters. (PDF 357 kb)
