# Supplementary material for: OrthoFinder: solving fundamental biases in whole genome comparisons dramatically improves orthogroup inference accuracy
Source: Genome Biol. 2015 Aug 6;16(1):157. doi: 10.1186/s13059-015-0721-2 (PMC4531804; doi:10.1186/s13059-015-0721-2)

Pairwise all-vs-all BLAST score transforms for Homo sapiens vs each other species

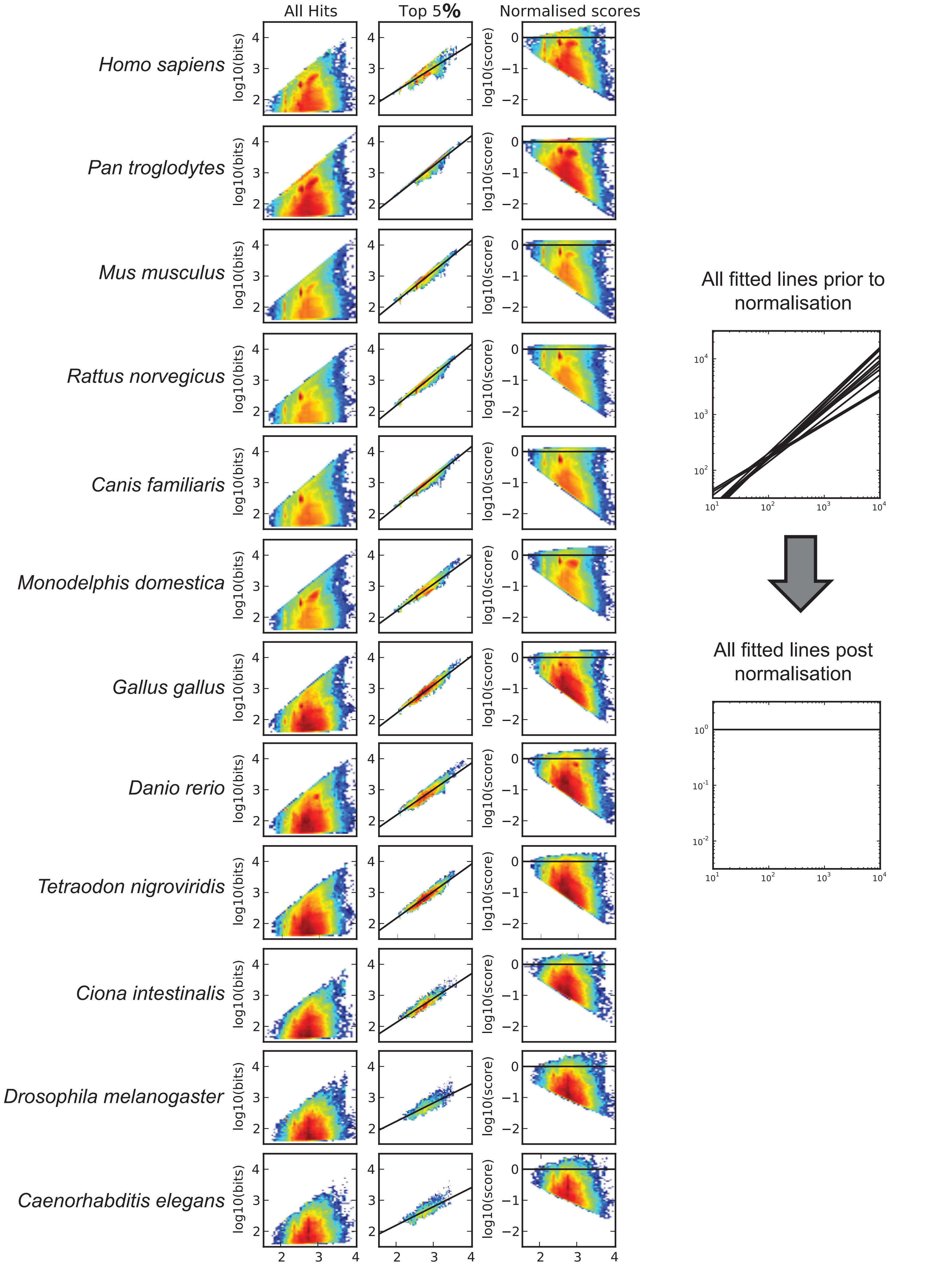

Supplement: Additional file 2: Figure S1. — An overview of how the OrthoFinder score transform also normalises for phylogenetic distance between BLAST scores. For illustration the All-Vs-All BLASTp scores are shown for the longest protein isoform from each protein coding gene in Homo sapiens vs all other species in the test. Note the difference in properties of fitted line, here the slope of the lines for the more closely related species are greater than for the more distant species. Following transform all fitted lines are transformed to the same value with a slope of 0. Thus the best scoring hits between distantly related species pairs and closely related species pairs achieve the same score hence normalising for interspecies phylogenetic distance. To provide further illustration all fitted lines between Homo sapiens and all other species before and after normalisation are shown on the right. (PDF 1440 kb) [file 13059_2015_721_MOESM2_ESM.pdf]
