# Supplementary figures and images for: OrthoFinder: solving fundamental biases in whole genome comparisons dramatically improves orthogroup inference accuracy
Source: Genome Biol. 2015 Aug 6;16(1):157. doi: 10.1186/s13059-015-0721-2 (PMC4531804; doi:10.1186/s13059-015-0721-2)

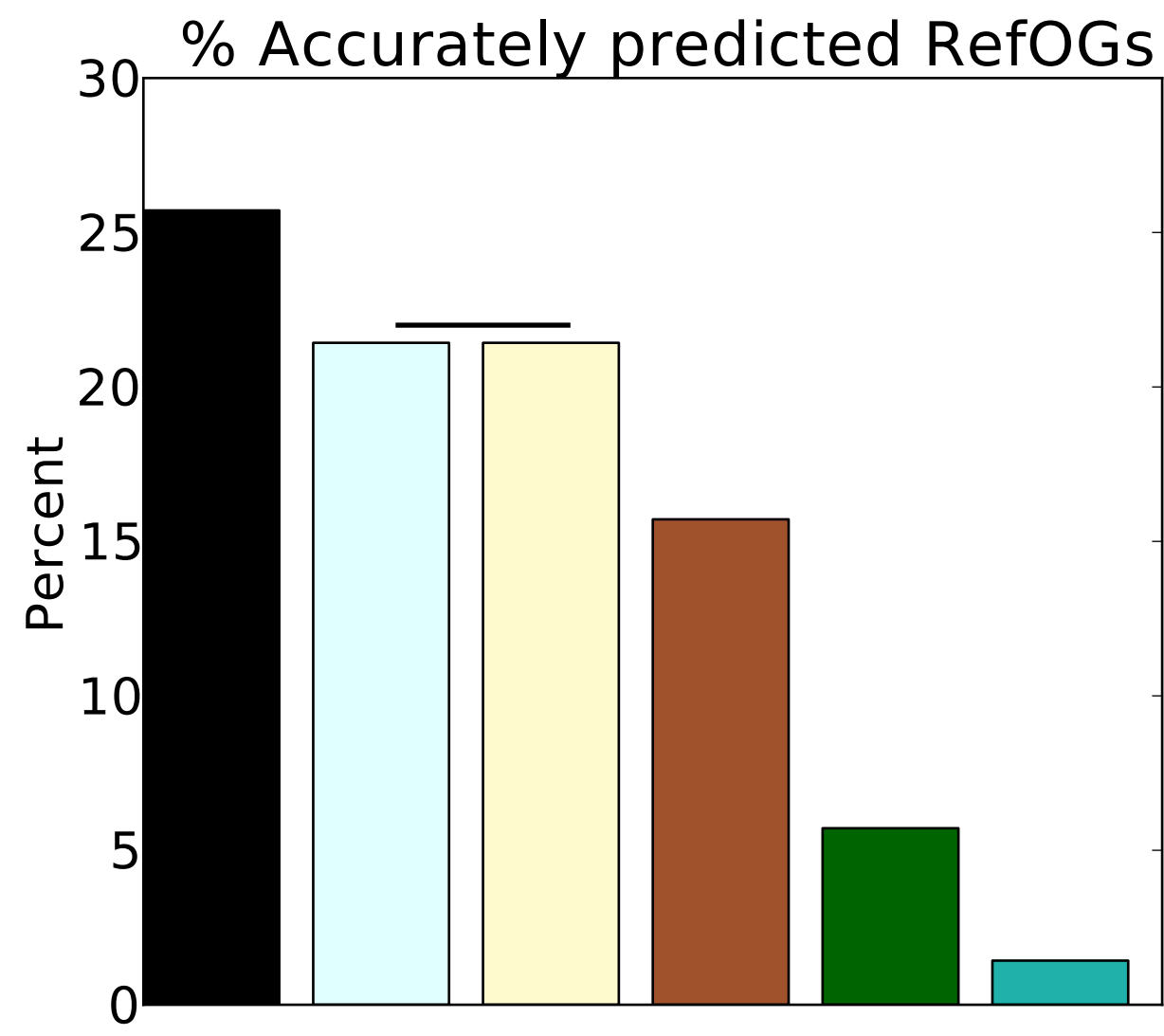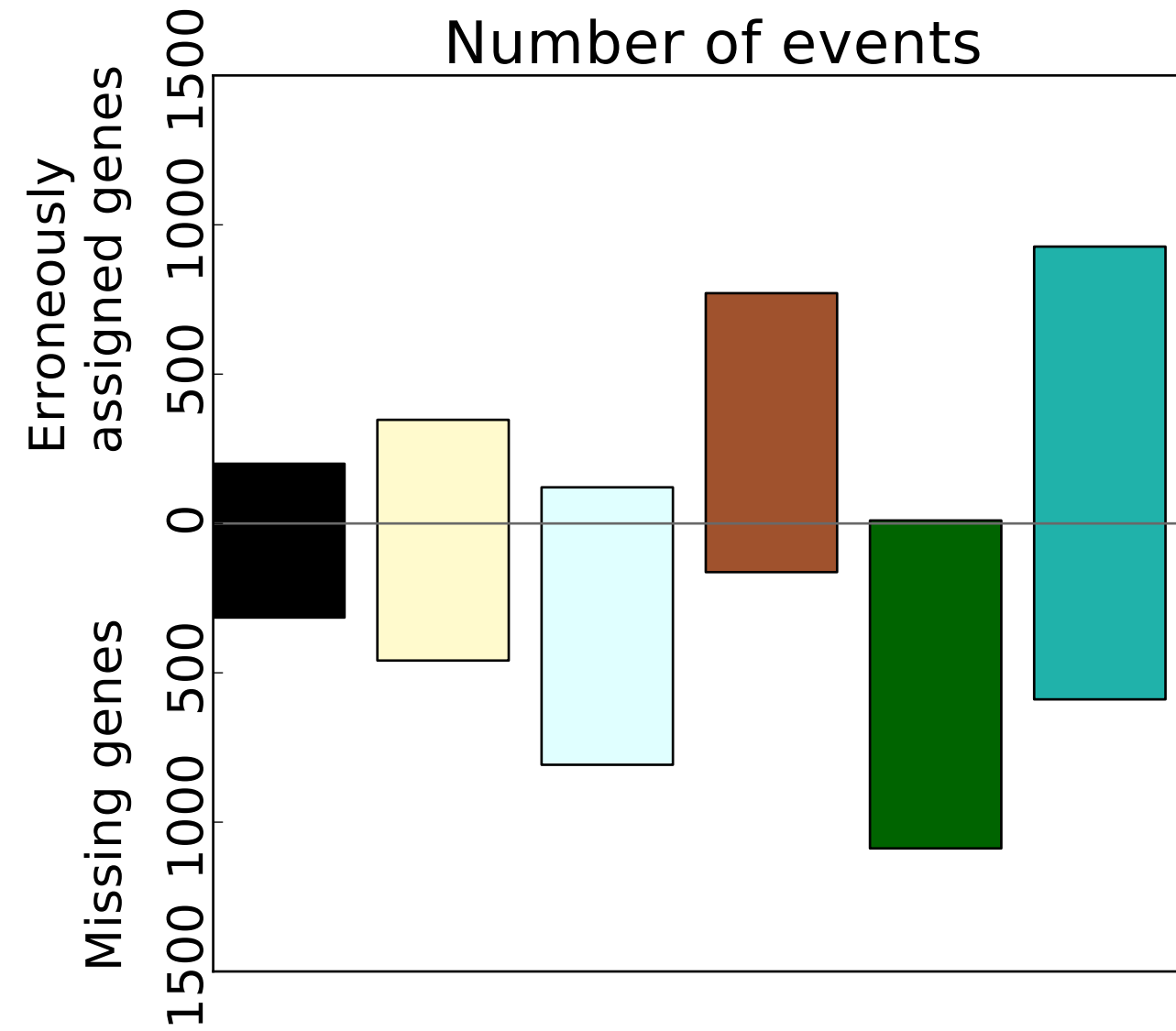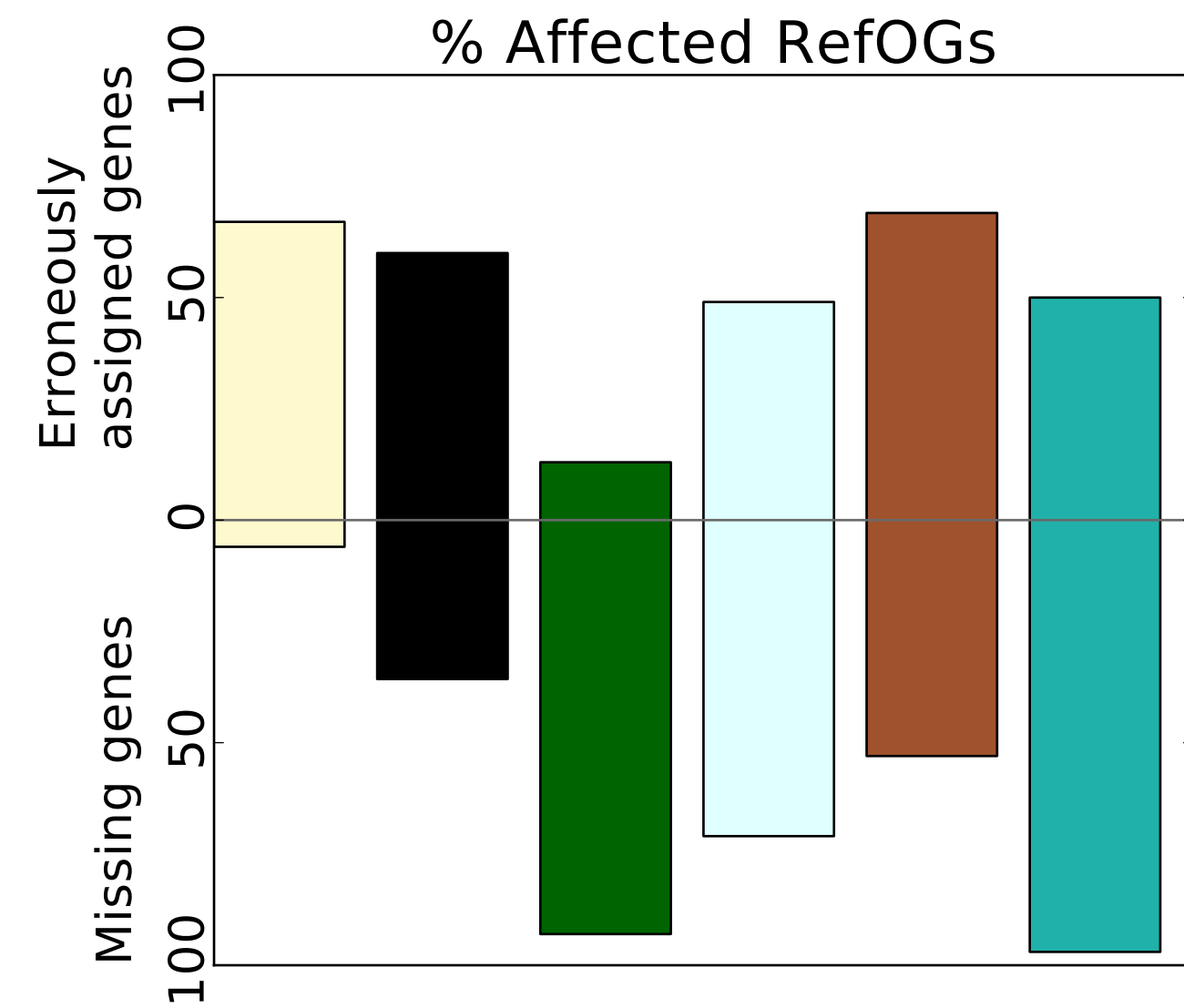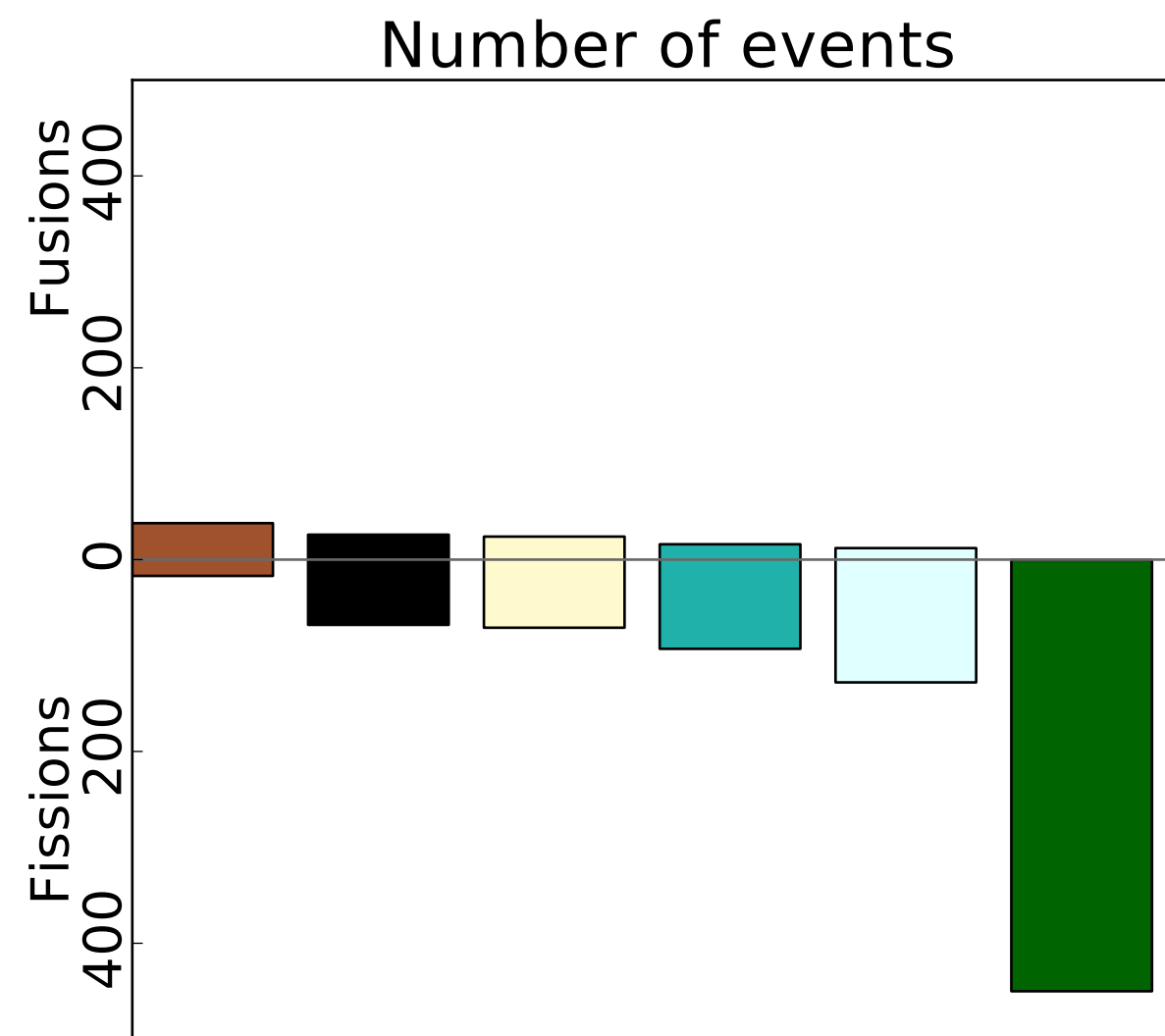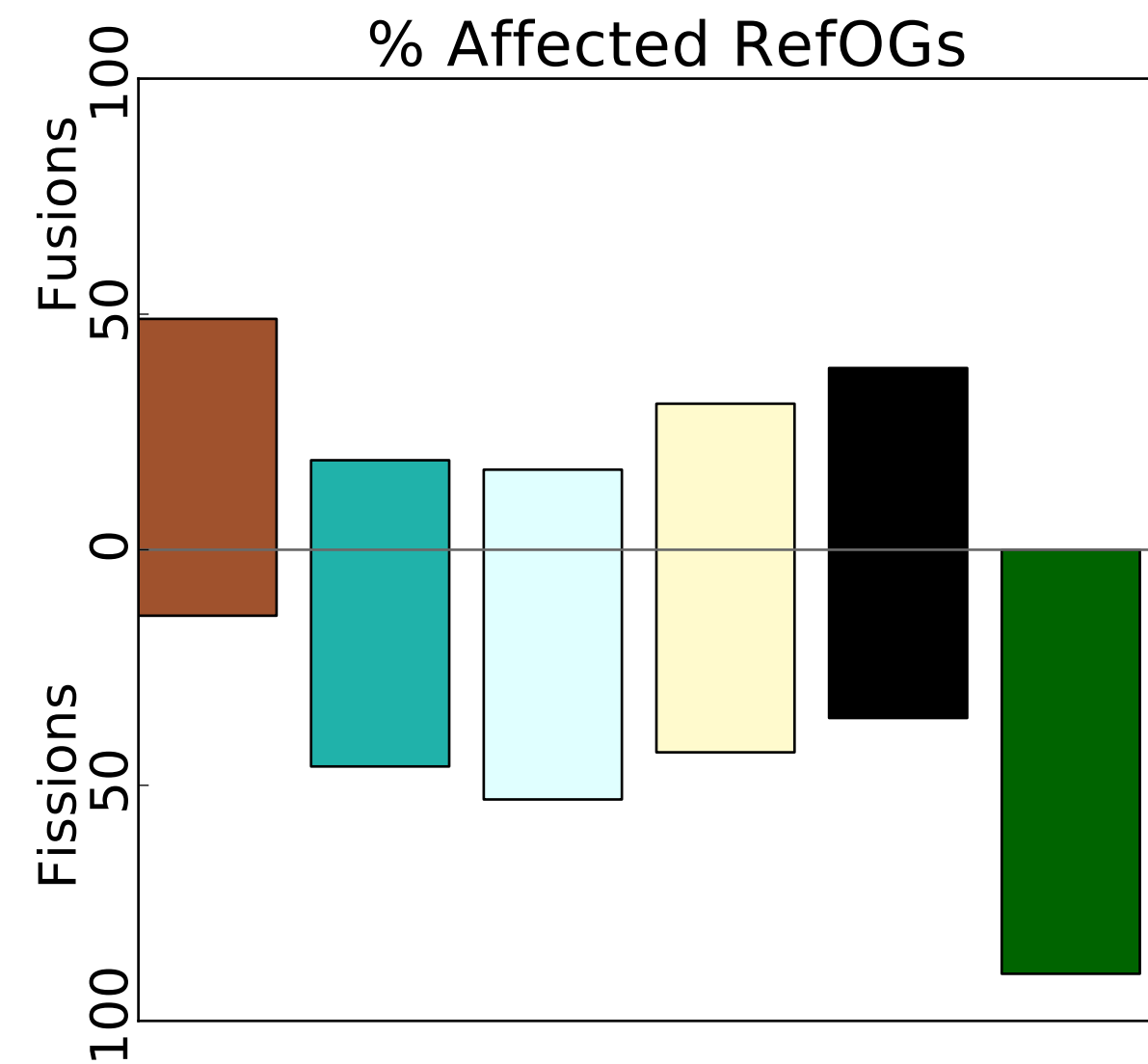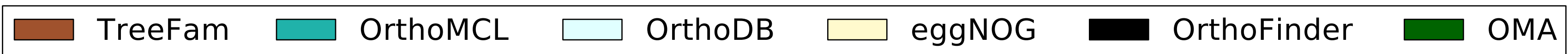

Supplement: Additional file 3: Figure S2. — Results on the OrthoBench dataset using additional assessment criteria presented in the original OrthoBench paper (the calculation for one of the plots could not be reproduced using the information provided in the OrthoBench paper and so has not been included). (PDF 29 kb) [file 13059_2015_721_MOESM3_ESM.pdf]

Random Families

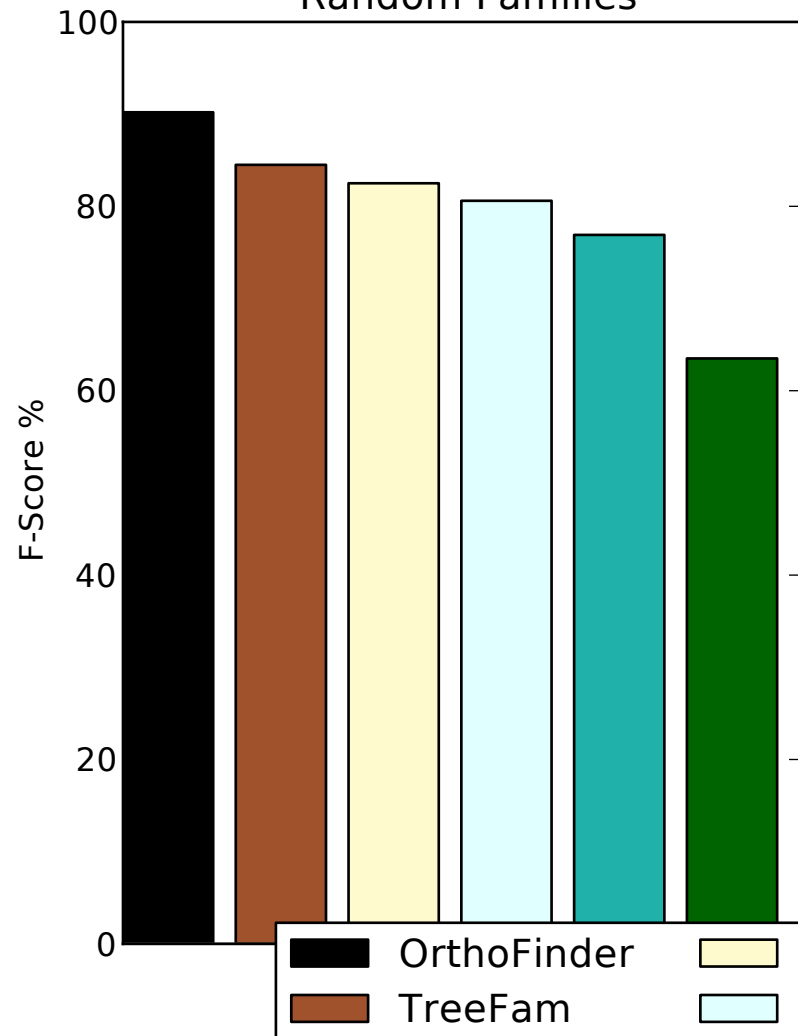

Challenging Families

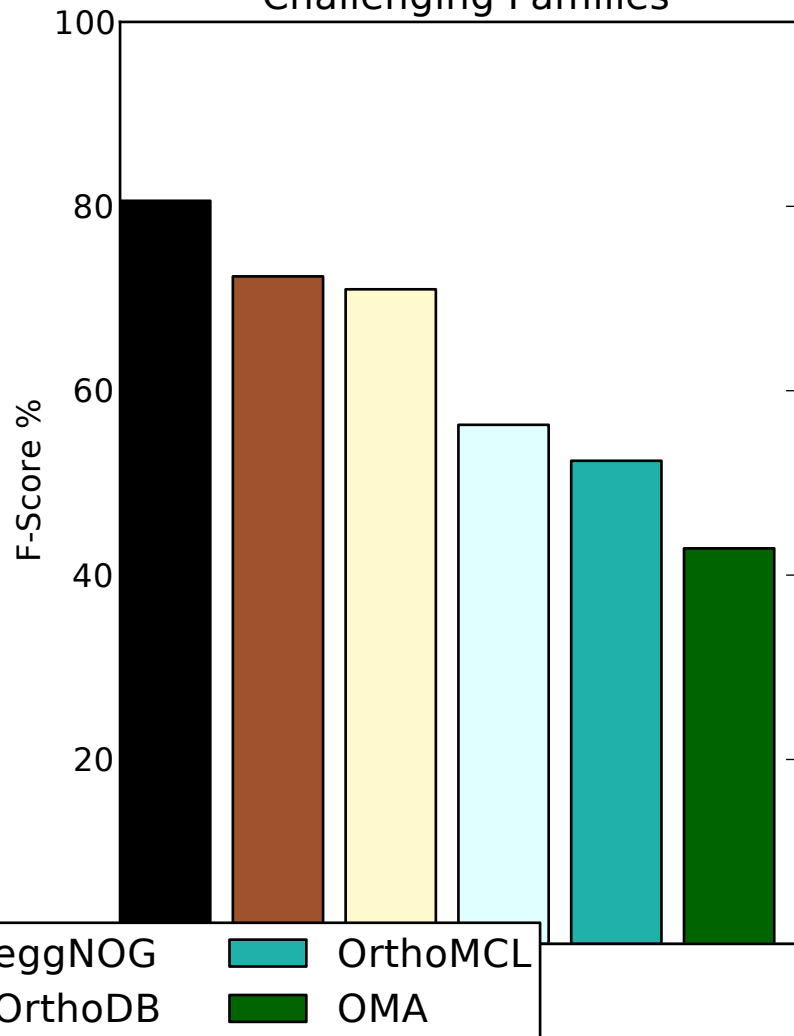

Supplement: Additional file 4: Figure S3. — F-scores on the OrthoBench dataset for the 30 randomly chosen gene families and the 40 biologically or technically challenging gene families that make up the dataset. (PDF 357 kb) [file 13059_2015_721_MOESM4_ESM.pdf]

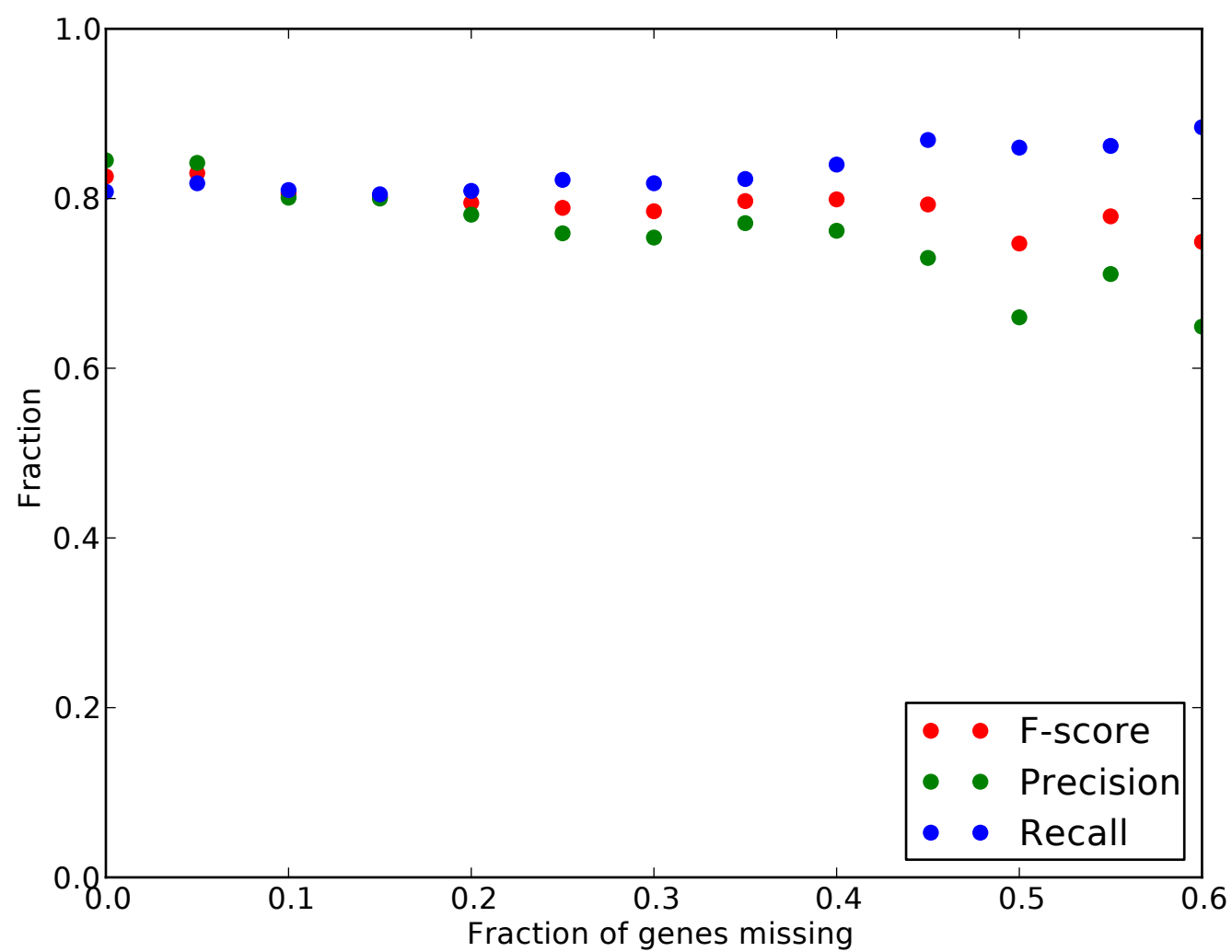

Supplement: Additional file 5: Figure S4. — Accuracy of OrthoFinder as a function of fraction of missing sequences. With poor gene coverage many RBNBs will be missing and so cannot inform the identification of orthogroups. To simulate this, genes were removed at random from the OrthoBench dataset input into the OrthoFinder and the precision, recall and F-score on the remaining genes were measured. (PDF 356 kb) [file 13059_2015_721_MOESM5_ESM.pdf]

# OrthoFinder

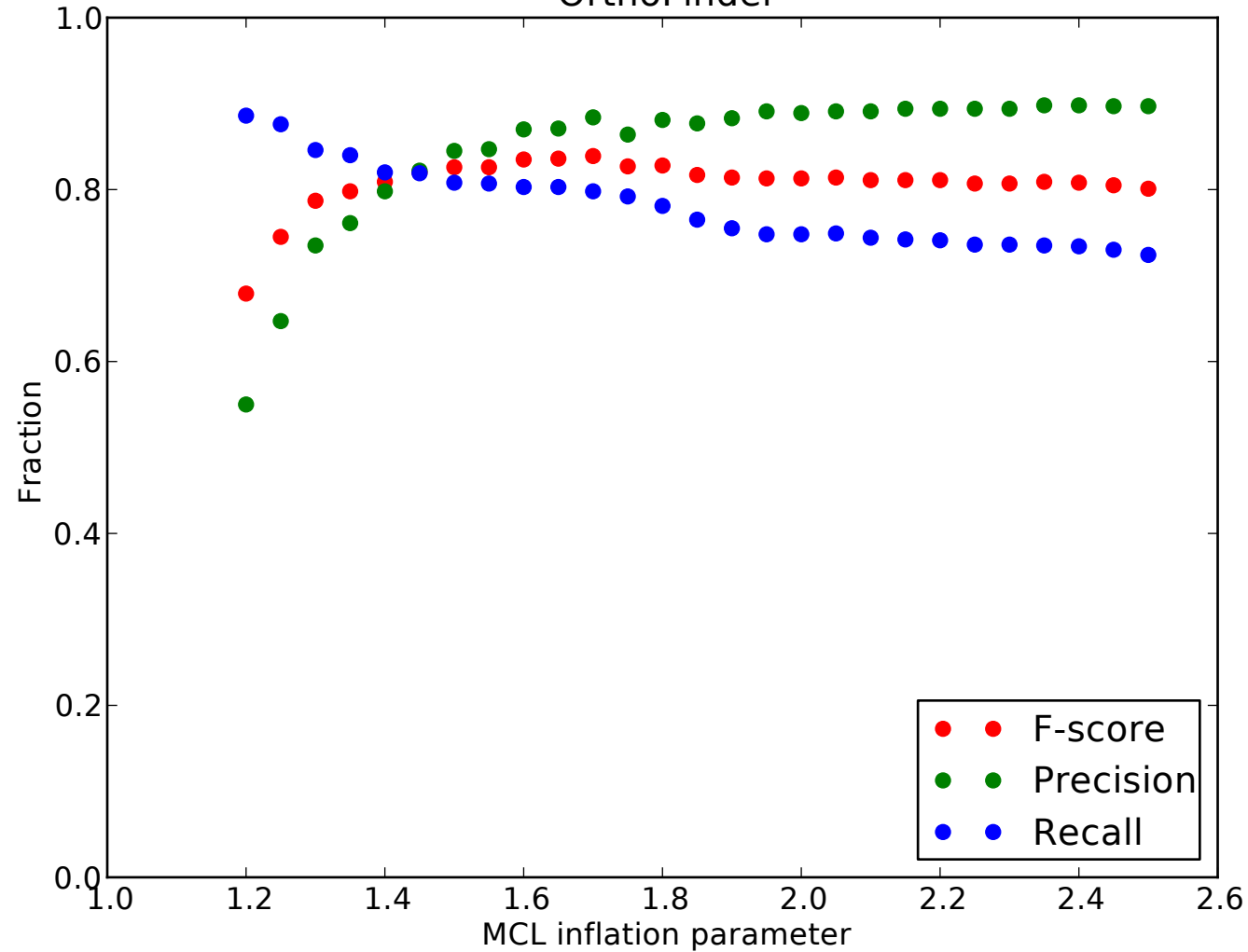

Supplement: Additional file 9: Figure S6. — The effect of the MCL inflation parameter on the F-score, precision and recall of OrthoFinder on the OrthoBench dataset. In OrthoFinder we use the default parameter of 1.5 which gives the results reported in this paper (84.5 %, 80.8 % and 82.6 % for precision, recall and F-score, respectively). Increasing the inflation parameter can be used to achieve higher precision at the cost of lower recall. Conversely, a smaller value of inflation can be used to achieve higher recall at the cost of lower precision. In this dataset the best result obtained by OrthoFinder in terms of F-score was 83.9 % using a value of 1.7 for the inflation parameter. The scores for precision and recall were 88.4 % and 79.8 %, respectively. (PDF 357 kb) [file 13059_2015_721_MOESM9_ESM.pdf]

# OrthoMCL

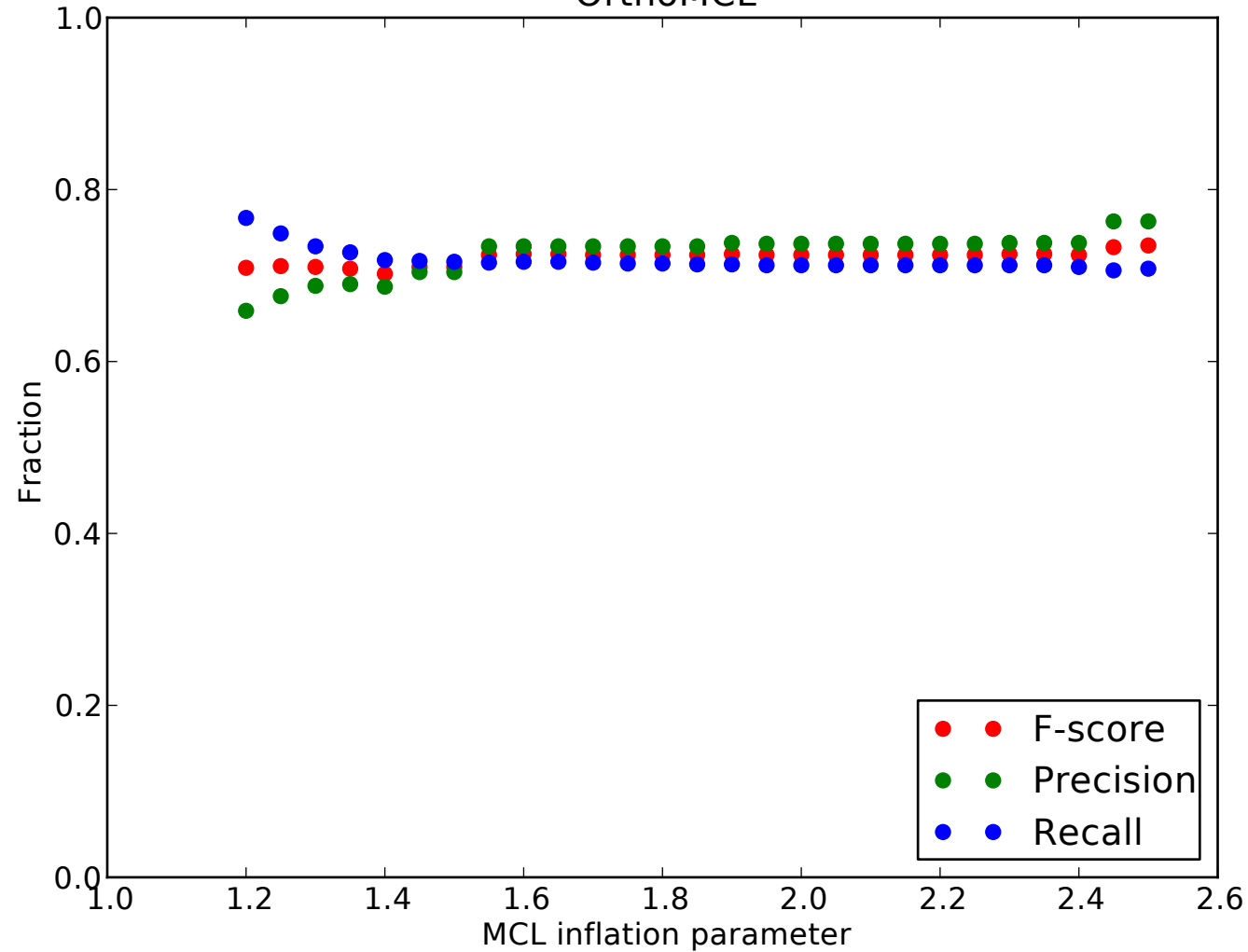

Supplement: Additional file 10: Figure S7. — The effect of the MCL inflation parameter on the F-score, precision and recall of OrthoMCL on the OrthoBench dataset. The standalone OrthoMCL v2.0.9 was run on the OrthoBench dataset to produce the MCL input graph and MCL was rerun on this graph with a range of inflation parameters. (PDF 357 kb) [file 13059_2015_721_MOESM10_ESM.pdf]
