# Supplementary material for: OrthoFinder: solving fundamental biases in whole genome comparisons dramatically improves orthogroup inference accuracy
Source: Genome Biol. 2015 Aug 6;16(1):157. doi: 10.1186/s13059-015-0721-2 (PMC4531804; doi:10.1186/s13059-015-0721-2)
Supplement: Additional file 8: Figure S5. — A worked example showing the criteria for identification of putative cognate gene-pairs used by OrthoFinder. (PDF 72 kb) [file 13059_2015_721_MOESM8_ESM.pdf]

Identification of pairwise gene relatioships prior to clustering into orthologous groups

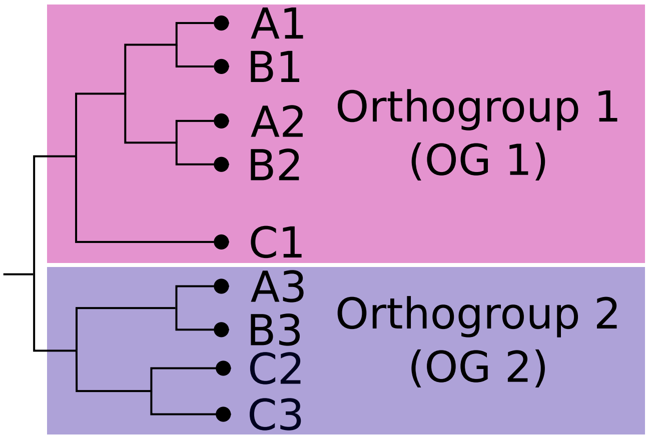

Assume for this worked example that similarity scores between genes decrease monotonically with time since divergence but that gene pairs with the same time since divergence will have different scores due to random variation. The RBBs will be,

OG1: `{A1,B1}, {A2,B2}, {Ai,C1}, {Bj,C1}`
where `i,j = 1 or 2` according to whether A1 or A2 has a higher similarity score with C1 and similarly for B1 and B2 with C1.

OG2: `{A3,B3}, {A3,Ci}, {B3,Cj}`
where `i,j = 1 or 2` (as explained above).

Without loss of generality we can consider two cases,
Case 1: `i=j` (use `i=j=1`),
Case 2: `i≠j` (use `i=1,j=2`).

OrthoFinder

Case 1

RBBs:
{A1,B1} {A3, B3}
{A2,B2} {A3, C3}
{A1,C1} {B3, C3}
{B1,C1}

Better hit than RBB:
{A1,A2} {C2,C3}
{A1,B2}
{B1,A1}
{B1,A2}

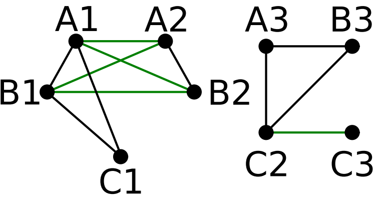

Case 2

RBBs:
{A1,B1} {A3,B3}
{A2,B2} {A3,C3}
{A1,C1} {B3,C3}
{B2,C1}

Better hit than RBB:
{A1,A2} {C2,C3}
{A1,B2}
{B2,A1}

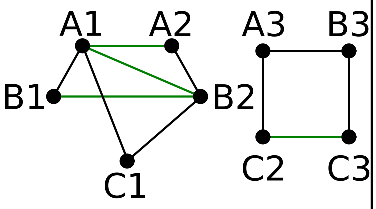

Proportions of cognate-pairs identified prior to clustering

OG 1: 8 / 10
OG 2: 4 / 6

OG 1: 7 / 10
OG 2: 4 / 6

OrthoMCL

Case 1

RBBs:
{A1,B1} {A3,B3}
{A2,B2} {A3,C3}
{A1,C1} {B3,C3}
{B1,C1}

Paralogues:
None {C2,C3}

Co-orthologues:
None {A3,C3}
{B3,C3}

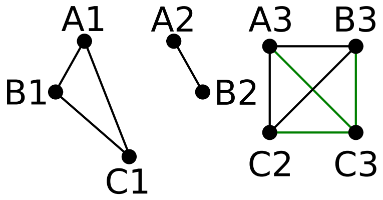

Case 2

RBBs:
{A1,B1} {A3,B3}
{A2,B2} {A3,C3}
{A1,C1} {B3,C3}
{B2,C1}

Paralogues:
None {C2,C3}

Co-orthologues:
None {A3,C3}
{B3,C2}

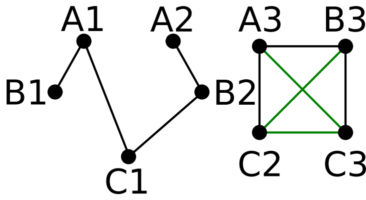

Proportions of cognate-pairs identified prior to clustering

OG 1: 4 / 10
OG 2: 6 / 6

OG 1: 4 / 10
OG 2: 6 / 6
